# Impact of anesthetic agents on the amount of bleeding during dilatation and evacuation: A systematic review and meta-analysis

**DOI:** 10.1371/journal.pone.0261494

**Published:** 2021-12-22

**Authors:** Hyun Ah Lee, Hiromasa Kawakami, Takahiro Mihara, Hitoshi Sato, Takahisa Goto

**Affiliations:** 1 Intensive Care Unit, Yokohama City University Medical Center, Yokohama, Kanagawa, Japan; 2 Operation Department, Yokohama City University Medical Center, Yokohama, Kanagawa, Japan; 3 Department of Anesthesiology, Yokohama City University Hospital, Yokohama, Kanagawa, Japan; 4 Department of Health Data Science, Graduate School of Data Science, Yokohama City University, Yokohama, Kanagawa, Japan; 5 Department of Anesthesiology, Yokohama City University Medical Center, Yokohama, Kanagawa, Japan; University of Phayao, THAILAND

## Abstract

**Purpose:**

Patients undergo dilatation and evacuation for abortion or miscarriage. However, bleeding is sometimes problematic. Despite reports on the association between volatile anesthetics and increased bleeding during the procedure, firm evidence is lacking. Therefore, we conducted a systematic review and meta-analysis to compare the effects of volatile anesthetics and propofol on the amount of bleeding in patients undergoing dilatation and evacuation.

**Methods:**

We conducted a systematic search of four databases, namely PubMed, Embase, Cochrane Central Register of Controlled Trials databases, and Web of Science (Clarivate Analytics), from their respective inception to April 2021. Moreover, we searched two trial registration sites. The inclusion criterion was randomized controlled trials of patients who underwent dilatation and evacuation under general anesthesia using volatile anesthetics or propofol. The primary outcome was the amount of perioperative bleeding. The mean difference of the bleeding was combined using a random-effects model. The I^2^ statistic was used to assess heterogeneity. We assessed risk of bias with Cochrane domains. We controlled type I and II errors due to sparse data and repetitive testing with Trial Sequential Analysis. We assessed the quality of evidence with GRADE.

**Results:**

Five studies were included in the systematic review. The amount of bleeding was compared in four studies and was higher in the volatile anesthetic group, with a mean difference of 164.7 ml (95% confidence interval, 43.6 to 285.7; p = 0.04). Heterogeneity was considerable, with an I^2^ value of 97%. Two studies evaluated the incidence of significant bleeding, which was significantly higher in the volatile anesthetic group (RR, 2.42; 95% confidence interval, 1.04–5.63; p = 0.04).

**Conclusion:**

Choosing propofol over volatile anesthetics during dilatation and evacuation might reduce bleeding and the incidence of excessive bleeding. However, the quality of the evidence was very low. This necessitates further trials with a low risk of bias.

**Trial registration:**

PROSPERO (CRD42019120873).

## Introduction

Dilatation and evacuation (D&E) or curettage is a short surgical procedure to remove uterine contents for induced abortion or to remove tissues that remain in the uterus following miscarriage. Volatile and intravenous anesthetics are widely used for the aforementioned procedure because of their fast onset and recovery.

D&E has low morbidity and mortality [1]. However, hemorrhage is one of the serious complications [2]. In some cases, the blood loss mounts up to 4000 ml [3], and patients may require additional procedures such as uterine artery embolization [4]. Even when hemorrhage is not massive, more bleeding may interfere with surgical condition and result in procedural difficulty or prolonged surgical duration. Advanced gestational age and indication for D&E have been associated with increased estimated blood loss during the procedure [3].

Postpartum hemorrhage is reportedly caused by uterine atony [5]. Furthermore, decreased uterine tone is associated with bleeding following abortion [6]. Thus, controlling uterine tone during the procedure may decrease bleeding-associated complications.

Some studies have demonstrated that volatile anesthetics might decrease the contractility of uterine muscles [7–11]. Propofol has also been found to decrease contractility to some extent [12]. Voltage-dependent calcium and potassium channels are involved in controlling the tone of pregnant uterine muscles and are affected by volatile anesthetics [11, 13].

Considering the varied effects of different anesthetics on uterine contractility [14], the choice of anesthetic agents can be associated with the amount of blood loss during D&E.

Recent retrospective studies report conflicting results regarding whether propofol use is associated with less hemorrhage compared to volatile anesthetics [3, 15]. There are several randomized clinical trials, however, the sample sizes in these trials are small. There has been no systematic review regarding the effect of volatile anesthetics for patients undergoing D&E. Thus, there is no current consensus on the most desirable agent for reducing hemorrhage during D&E.

We conducted a systematic review and meta-analysis of randomized clinical trials (RCTs) that compared propofol and volatile anesthetics during D&E. The relationship between the choice of anesthetic agents and the amount of blood loss during the procedure was the primary outcome.

## Materials and methods

We followed the Preferred Reporting Items for Systematic Reviews and Meta-Analyses (PRISMA) statement [16] and the Cochrane Handbook [17]. Our study protocol and methods were pre-specified and registered on PROSPERO (CRD42019120873). They can be accessed at https://www.crd.york.ac.uk/prospero/display_record.php?RecordID=120873.

### Search strategy

We searched four databases, namely PubMed, EMBASE, Cochrane Central Register of Controlled Trials (CENTRAL), and Web of Science. The last search was conducted on April 19, 2021. We also searched trial registration sites, such as ClinicalTrials.gov and the University Medical Information Network Trial Registry. Our search strategy included terms only related to the procedure and intervention. The search strategy for PubMed has been attached (S1 Text). There were no language restrictions.

### Inclusion criteria

We searched for RCTs that compared volatile anesthetics with propofol as agent for anesthesia maintenance for patients who underwent a surgical termination of pregnancy or D&E for miscarriage. We excluded studies that did not evaluate blood loss. In addition, we excluded data from case reports, observational studies, comments, letters to the editor, reviews, and animal studies.

Three authors (H. K., H. L., and H. S.) independently screened the titles and abstracts of the retrieved studies to identify those that potentially met the inclusion criteria. The full texts of these potentially eligible studies were retrieved and independently assessed for their eligibility by two authors (H. K. and H. L.). Moreover, the complete article was also retrieved if the eligibility could not be determined from the title or abstract.

### Outcomes

The amount of blood loss during the procedure was the primary outcome. In contrast, the number of patients with excessive bleeding was the secondary outcome. Additional outcomes included surgical duration, the subjective measurement of surgical difficulty, and adverse events, such as postoperative nausea and vomiting.

### Data collection

We created a data collection sheet that included the following parameters: (1) the American Society of Anesthesiologist physical status, (2) age, (3) exclusion criteria, (4) gestational age, (5) the type of induction agent, (6) the type of maintenance agent, (7) the number of patients in the group, (8) the amount of intraoperative bleeding, (9) surgical difficulty, (10) the number of patients with excessive intraoperative bleeding, (11) the number of patients with excessive postoperative bleeding, (12) the duration of surgery and (13) adverse events such as postoperative nausea and vomiting, length of post-anesthesia care unit stay, or duration until hospital discharge.

### An assessment of the risk of bias

We followed the Cochrane Handbook for Systematic Reviews of Interventions to assess the risk of bias [18]. Two authors (H.L., H.K.) evaluated the sequence generation, allocation sequence concealment, the blinding of patients and outcome assessors, incomplete outcome data, selective reporting, and other biases in each trial. Subsequently, the risk of bias was classified into three categories as follows: (1) low, (2) high, or (3) unclear. Trials with one or more unclear or high risks of bias were considered having an overall high risk of bias.

### Assessing the quality of evidence

We graded the quality of evidence of the outcomes according to the Grading of Recommendations Assessment, Development, and Evaluation (GRADE) approach, based on the risk of bias, inconsistency, indirectness, the imprecision of results, and publication bias [19, 20]. We formulated a summary of findings table using GRADEpro GDT (https://gradepro.org/). The GRADE was categorized as very low, low, moderate, or high.

### Statistical analyses

We conducted the meta-analysis using Review Manager software (RevMan version 5.3; The Nordic Cochrane Centre, Copenhagen, Denmark). Continuous data were summarized using a mean difference with a 95% confidence interval (CI) to assess the presence of a significant difference in the amount of blood loss between the volatile anesthetic and propofol groups. We estimated the mean and standard deviation using the method by Wan et al. for a study that reported on the median and range of continuous data [21]. The difference was considered statistically insignificant if the 95% CI included a value of 0. Moreover, we conducted a risk ratio analysis with 95% CI for studies that reported on excessive bleeding. The difference was considered insignificant if the 95% CI included a value of 1. The random effects model (Dersimonian and Laird method [22]) was used to combine the results. Heterogeneity was quantified using I^2^ statistics.

While an I^2^ value ranging between 30–60 indicated moderate heterogeneity, one >75 indicated considerable heterogeneity [17]. Forest plots were used to evaluate and depict the effects of the studies. We evaluated the small study effect, a tendency for the intervention effects estimated in smaller studies to be larger, using a funnel plot and Egger’s regression asymmetry test [23] for sufficient number of studies (>10). The asymmetrical funnel plot suggested the possibility of a small study effect.

Sensitivity analysis was performed for the primary outcome according to the risk of bias to evaluate the robustness of the results. The risk of bias was evaluated as low or high.

There is a risk of random errors in the conventional meta-analysis due to repetitive testing of accumulating and sparse data. We conducted a Trial Sequential Analysis (TSA) to correct for the increased type 1 error. We predetermine the risk of type 1 error, statistical power, and clinically significant difference in the amount of blood loss. And then the Z-cumulative curve, and the trial sequential boundaries can be drawn. We can also calculate the required information size (RIS), the required number of patients to decide whether the difference exists or not. The risk of type 1 error was maintained at 5%, with a power of 90%, and we predetermined the clinically significant difference in blood loss of 100 ml. The Z-cumulative curve crossing the trial sequential monitoring boundary for the futility on the graph suggested adequate evidence to conclude that the difference was not greater than the predetermined clinically significant difference of 100 ml. If the Z-cumulative curve crosses the trial sequential boundary for benefit, it is considered that sufficient data have been accumulated to demonstrate that the difference is significant. TSA was performed using TSA viewer version 0.9.5.10 Beta. (www.ctu.dk/tsa).

## Results

**Fig 1:**
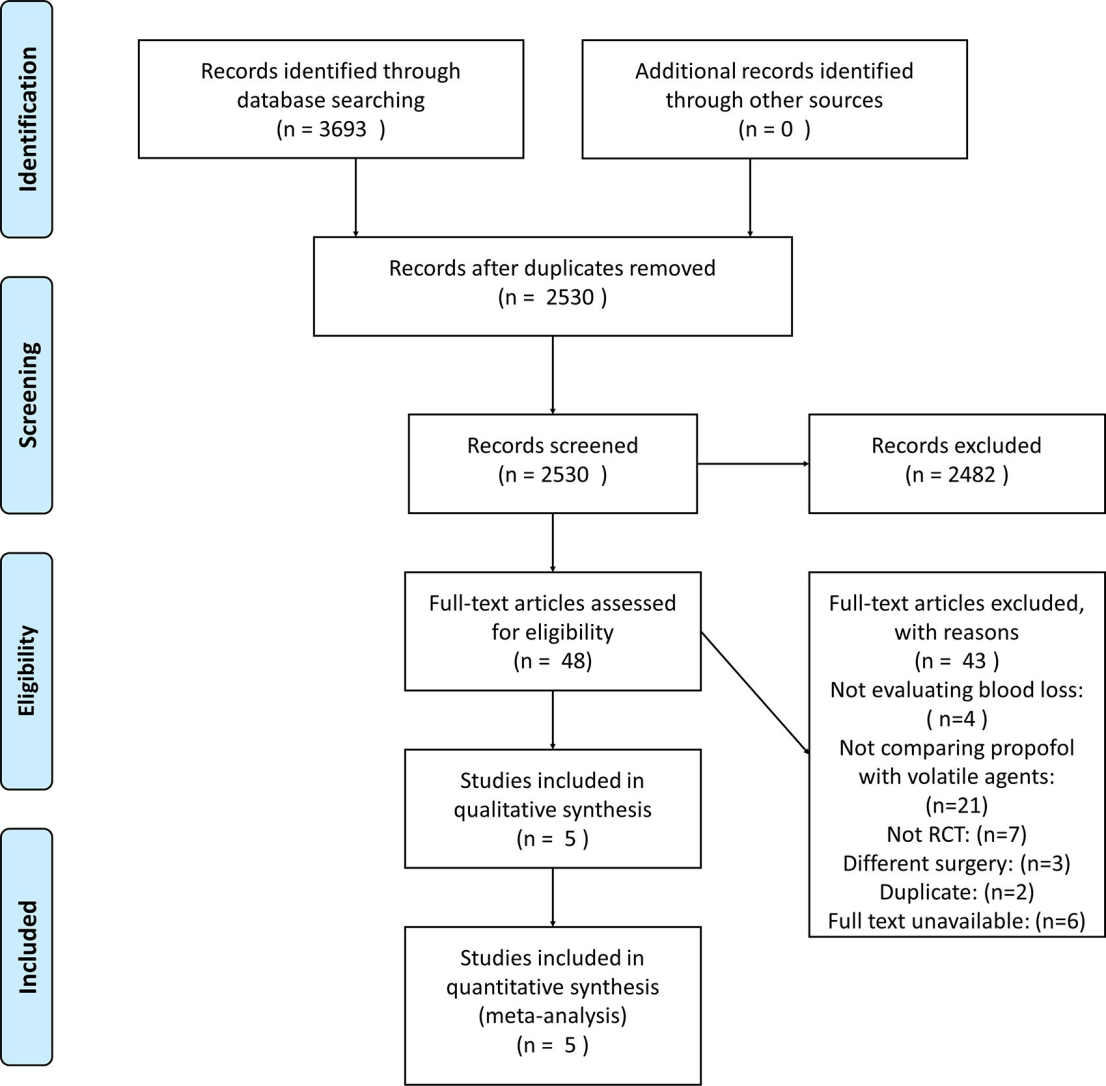
PRISMA flow diagram.

A total of 3,693 publications were identified. Of these studies, five were included in this review [24–28]. Fig 1 depicts the PRISMA flow diagram detailing the disposition of retrieved publications. The evaluated trials included data from 414 subjects, and 208 of them received inhalational anesthetics.

Table 1 summarizes the features of the included RCTs. All but one trial included patients who underwent a termination of pregnancy. While three trials compared isoflurane with propofol, the remaining two trials compared sevoflurane with propofol.

**Table 1:** Study characteristics.

Source	ASA-PS	age (protocol)	gestational age (protocol)	indication for surgery	Study Drug	induction agent for volatile group
Kumarasinghe 1997	1–2	not reported	6–14 weeks	termination of pregnancy	isoflurane	propofol
Hall 1997	1–2	not reported	9-12weeks	termination of pregnancy	isoflurane	propofol
Nathan 1998	1	>18yr	not reported	termination of pregnancy	sevoflurane	sevoflurane
Nelskyla 1999	not reported	>18yr	<12 weeks	termination of pregnancy or miscarriage	sevoflurane	sevoflurane
Micks 2015	not reported	>16yr	18–24 weeks	termination of pregnancy	sevoflurane	propofol

ASA-PS, American Society of Anesthesiologists-Physical Status.

### Risk of bias of the included trials

Table 2 summarizes the risks of bias. While one trial [27] was considered at a low risk of bias, the rest were considered at a high risk of bias. Nathan et al. terminated the trials because of unpredictable variations of perioperative uterine bleeding in the volatile group [26].

**Table 2:** Risk of bias of each trial.

Source	Sequence generation	Allocation concealment	Patients blinded	Outcome assessors blinded	Incomplete outcome data	Selective reporting	Other bias	Summary
Kumarasinghe 1997	Low	Low	Unclear	Low	Low	Low	Low	High
Hall 1997	Unclear	Low	Unclear	Unclear	Low	Low	Low	High
Nathan 1998	Unclear	Low	Unclear	Unclear	High	Low	Low	High
Nelskyla 1999	Unclear	Low	Unclear	Low	Low	Low	Low	High
Micks 2015	Low	Low	Low	Low	Low	Low	Low	Low

### The amount of blood loss

**Fig 2:**
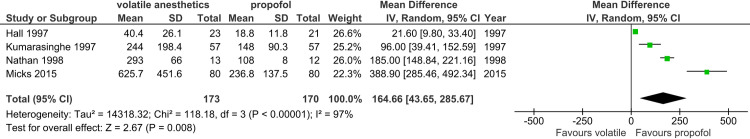
Pooled data evaluating amount of bleeding.

**Fig 3:**
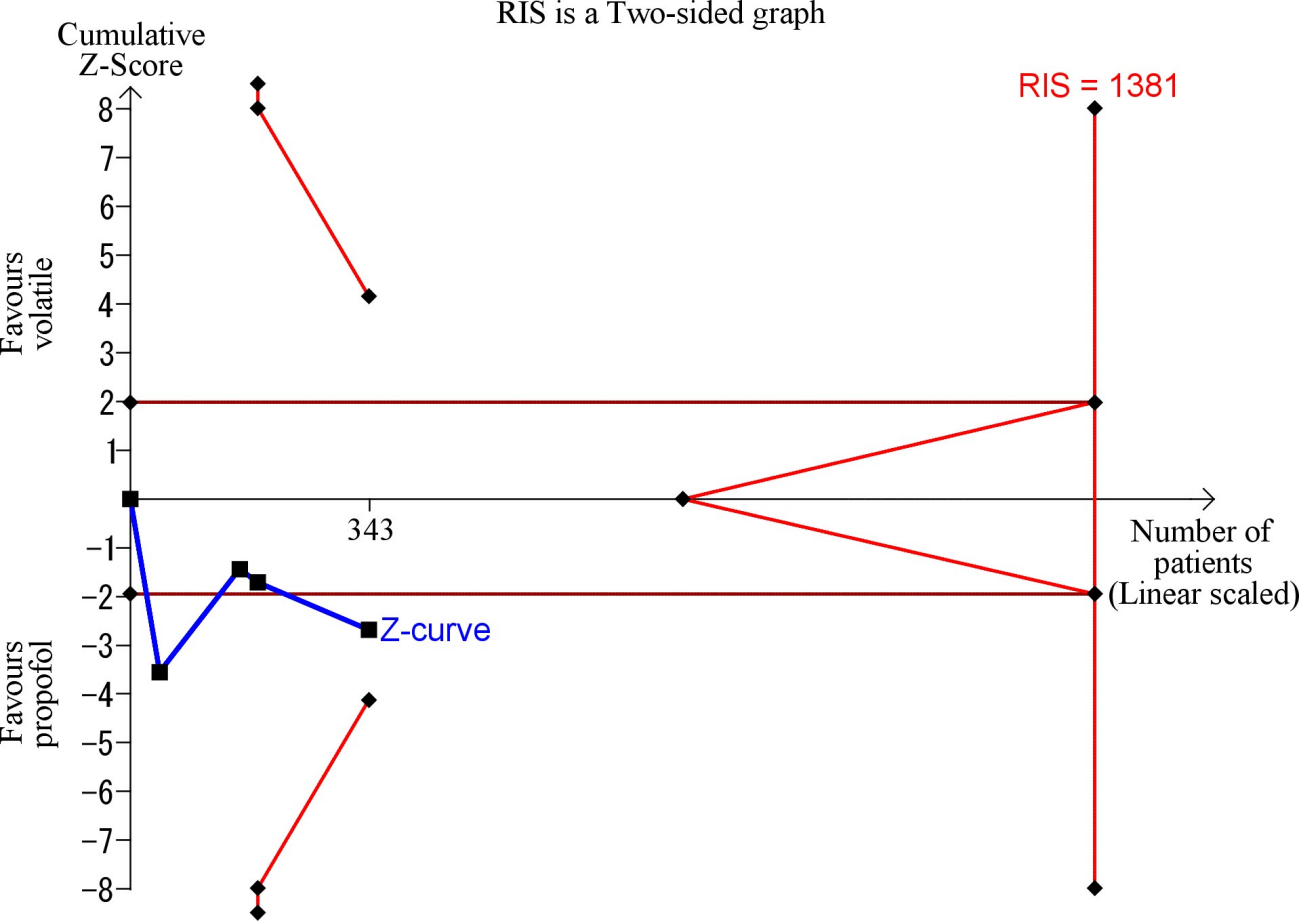
Trial sequential analysis for determining the effect of volatile anesthetics on the amount of bleeding in patients undergoing dilatation and evacuation compared to propofol.

Three trials reported on the amount of intraoperative bleeding [24, 25, 27], and one trial evaluated the sum of intraoperative and postoperative hemorrhage [26]. Kumarasinghe et al. reported on the amount of bleeding with mean and 95% CI [24]. We calculated the SD from 95% CI and the number of patients [24]. Hall et al. calculated the amount of bleeding from the hemoglobin concentration of the patients and the iron content of the operative suction sample [25]. Nathan et al. measured the amount of intraoperative bleeding in half of the recruited patients [26]. Two manuscripts reported on the volume of bleeding [24, 27]. Moreover, the weight of uterine aspiration was reported in one trial [26]. We considered the uterine aspiration during the procedure to be the sum of fetal component and bleeding. Since the fetal component should not be affected by the anesthetic agents, we assumed the difference in the weight between the groups was because of blood and converted the weight into volume assuming the specific gravity of the patient’s blood was 1.05 g/ml. Fig 2 outlines the combined results. Patients receiving propofol for the maintenance of anesthesia had significantly less bleeding than those receiving volatile anesthetics (MD 164.7 ml; 95% CI, 43.6–285.67; p = 0.008). There was considerable heterogeneity, with an I^2^ of 97%. The TSA revealed that the accrued information size (n = 343) reached only 24.8% of the estimated RIS (n = 1381) (Fig 3). Moreover, the cumulative Z score did not cross the trial sequential boundaries, suggesting sufficient data had not been accumulated.

The risk of type 1 error has been maintained at 5%, with a power of 90%. The variance has been calculated from the data obtained from the included trials. A clinically significant difference in the amount of bleeding has been set to 100 ml. Red lines on the left denote the trial sequential boundaries for efficacy (above) or harm (below). While blue lines denote the Z-statistics for cumulative meta-analyses, each black dot on the blue lines represents one trial. The inner part of the red lines at the far right indicates the futility region. Horizontal green lines denote the efficacy and harm boundaries if no adjustment was made for repeated testing over time. Moreover, they represent a Z score of +1.96 and −1.96, indicating a conventional significant P-value of .05. The cumulative Z-curve has not crossed the trial sequential boundary for efficacy. The RIS indicates the required information size.

The incidence of excessive bleeding was recorded in two trials. We combined the incidence of blood loss >300 ml [27] and abundant bleeding [28]. The incidence of excessive bleeding was significantly higher in patients receiving volatile anesthetics (RR, 2.42; 95% CI, 1.04–5.63; p = 0.04) (S1 Fig).

### Surgical difficulty

While surgical difficulty was evaluated in one trial [27], gynecologists’ satisfaction was evaluated in another [28]. There were no significant differences between the groups. The procedure time was evaluated in two trials [25, 27], and there was no difference between the groups (MD 0.16; 95% CI, -0.61–0.94; p = 0.26) (S2 Fig).

### Adverse events

Postoperative nausea and vomiting were evaluated in 3 trials [26–28]. The incidence of nausea and vomiting was higher in the volatile group, but the difference was not significant (RR 3.00; 95% CI, 0.88–10.21; p = 0.08; I^2^ = 73%) (S3 Fig). Length of hospital stay was recorded in only one trial [28] as home readiness, and there was no significant difference.

### Sensitivity analysis

We did not perform a sensitivity analysis for the primary outcome. This is because only one of the four trials was at a low risk of bias. All trials reported on larger amount of blood loss during surgery in the volatile anesthetics group.

### Small study effect

We did not perform an asymmetry test for the funnel plot, owing to the inclusion of only four trials.

### Quality of evidence

The quality of evidence for the effect of volatile anesthetics on the amount of bleeding during dilatation and curettage as compared with propofol was graded as “very low” (S1 Table). The downgraded quality can be attributed to the limited study design, inconsistency, imprecision, and possible publication bias.

## Discussion

Our findings demonstrated that the use of volatile anesthetics tends to be associated with increased blood loss compared to propofol. However, this result should be cautiously interpreted. Despite the traditional meta-analysis revealing a significant difference, the TSA demonstrated that the difference was insignificant, and sufficient data have not been accumulated. Moreover, the quality of evidence was very low.

The mean difference in blood loss volume was 164.7 ml, and the incidence of excessive bleeding was significantly higher in patients who received volatile anesthetics. The mean amount of bleeding was higher in the volatile anesthetics group than that in the propofol group. However, the difference varied considerably. The mean difference in the amount of bleeding was greater than the clinically significant difference for TSA. A 100-ml difference in blood loss was not likely to change hemodynamic management. Nonetheless, the aforementioned difference may cause surgical difficulty, prolong the surgery, and affect the satisfaction of clinicians.

The difference in the amount of bleeding was extremely small in the trial by Hall et al. [25], in which the amount of bleeding following propofol administration was much smaller than that in the rest of the trials. They reported on the surgical duration without a significant difference. Moreover, the clinical significance of a higher amount of bleeding in the volatile anesthetic group was minimal.

Studies that evaluated the surgical difficulty [27] and satisfaction of gynecologists [28] reported no significant differences between the groups. There was no significant difference in the duration of the procedure. Avoiding inhalational agents for D&E may be reasonable for a high baseline risk of bleeding.

We downgraded the GRADE because of the study design, inconsistency, imprecision, and possible publication bias. Only one study was evaluated to have a low risk of bias. Studies at a high risk were published more than 20 years ago. The study protocols were not sufficiently systematic to meet the current standards, and the details of the protocol were missing, such as sequence generation and allocation concealment. There was significant heterogeneity among the studies. The inclusion of only five studies creates the possibility of a small study effect.

The I^2^ of the primary outcome was 97%, and heterogeneity among the trials was considered significant. This might be partly attributed to the inhomogeneous effect of volatile anesthetics. Two types of volatile anesthetics were used in the trials, namely isoflurane and sevoflurane. We designed this review on the assumption that volatile anesthetics would exert a similar effect on uterine muscles than propofol. However, there might be no ignorable differences in the effect even among the volatile ones. One study demonstrated that the inhibitory potency of isoflurane was less prominent than that of halothane. However, the potency of sevoflurane and desflurane was comparable to that of halothane [7]. In addition, the concentration of volatile anesthetics ranged from 1 to 1.5 MAC, depending on the trials. Sevoflurane and isoflurane reportedly decrease uterine contractility in a concentration-dependent manner [7, 13]. Thus, variety in the concentration of volatile anesthetics among the trials may have resulted in heterogeneity.

The amount of bleeding in the propofol group differed among the trials. Advanced gestational age is a risk factor for bleeding after abortion [1, 3]. Furthermore, the gestational age of the patients differed and the means of measuring blood loss differed among the trials. Surgical technique may affect blood loss, and Kittiwatanakul et al. have reported that electric vacuum aspiration is associated with less blood loss compared with sharp curettage [29]. Experience or skill can be associated with the outcome, and medications can also reduce perioperative blood loss. Misoprostol has been reported to reduce bleeding after surgery for spontaneous abortion [30]. The aforementioned factors might have resulted in heterogeneity.

Uterine atony is reportedly associated with hemorrhage after abortion [6]. Researchers have not yet established the effect of uterotonics as prophylaxis for blood loss after D&E. Kerns et al. demonstrated that methylergonovine prophylaxis after D&E for gestational age of 20 to 24 weeks did not decrease excessive postoperative bleeding [31]. Whitehouse et al. demonstrated that the prophylactic administration of oxytocin to patients undergoing D&E at 18 to 24 weeks gestation decreased blood loss and shortened their surgical duration [32]. In contrast, another RCT revealed that prophylactic oxytocin exerted no effect on postoperative vaginal bleeding in patients undergoing D&E during their first trimester [33].

Despite the conventional meta-analysis revealing a statistically significant difference between volatile anesthetics and propofol, the TSA demonstrated that the difference was insignificant, thus necessitating additional data to reach a conclusion. We picked up 100 ml for the meaningful difference in blood loss. We could not find any previous reports that used a particular amount for clinically meaningful differences for this kind of procedure. We expected that the mean blood loss in the volatile anesthetic group would be a few hundred ml, and a 20 to 40% reduction in blood loss would be considered clinically meaningful. Even though the difference of 100 ml might not change hemodynamic management, we speculated that this difference might change the surgical difficulty.

Spinal anesthesia is an anesthetic option for this procedure. It has been reported that gastric emptying is not delayed in pregnant patients not in labor [34, 35]. In our practice, patients go home on the day of surgery and spinal anesthesia is usually avoided. We could not find any studies that examined whether spinal anesthesia affects blood loss during or after dilatation and curettage.

Our study had several limitations. First, only five RCTs were found suitable for the analysis. The number of patients did not reach the RIS. Thus, the cumulative data failed to reach a sufficient level to determine the difference. Four studies were published more than 20 years ago, and the patient management or procedure may be different. Second, only one trial was evaluated to be at low risk of bias. Moreover, we could not deny the possibility of publication bias. In addition, there was significant heterogeneity among the trials. Therefore, the quality of evidence in this meta-analysis was very low.

In conclusion, choosing propofol over volatile anesthetics during D&E might reduce bleeding and the incidence of excessive bleeding. However, the quality of the evidence was very low, thus necessitating further trials with a low risk of bias.

## Supporting information

S1 Checklist PRISMA checklist. (DOC)

S1 Text Search strategy for PubMed. (PDF)

S1 Fig Pooled data evaluating incidence of excessive bleeding. (PDF)

S2 Fig Pooled data evaluating surgical duration. (PDF)

S3 Fig Pooled data evaluating postoperative nausea and vomiting. (PDF)

S1 Table Summary of findings. (PDF)

S1 Protocol Preregistered protocol for PROSPERO. (PDF)
