# Supplementary material for: Impact of anesthetic agents on the amount of bleeding during dilatation and evacuation: A systematic review and meta-analysis
Source: PLoS One. 2021 Dec 22;16(12):e0261494. doi: 10.1371/journal.pone.0261494 (PMC8694452; doi:10.1371/journal.pone.0261494)
Supplement: S1 Text — (PDF) [file pone.0261494.s002.pdf]

Pubmed

("dilatation and curettage"[mh] OR (dilatation[tiab] AND curettage[tiab]) OR (dilatation[tiab] AND evacuation[tiab]) OR (vacuum[tiab] AND curettage[tiab]) OR (suction[tiab] AND curettage[tiab]) OR (uterine[tiab] AND aspirat*[tiab]) OR "abortion, spontaneous"[mh] OR abortion[tiab] OR miscarriage[tiab] OR "early pregnancy loss"[tiab] OR (termination[tiab] AND pregnancy[tiab])) AND (anesthesia[mh] OR anesthesia[tiab] OR anaesthesia[tiab] OR propofol[mh] OR propofol[tiab] OR sevoflurane[mh] OR sevoflurane[tiab] OR isoflurane[mh] OR isoflurane[tiab] OR desflurane[mh] OR desflurane[tiab] OR anesthetics[Pharmacological Action] OR anesthetics[mh] OR anesthetics[tiab] OR anesthesiology[mh] OR anesthesiology[tiab]) AND (randomized controlled trial [pt] OR controlled clinical trial [pt] OR randomized [tiab] OR placebo [tiab] OR drug therapy [sh] OR randomly [tiab] OR trial [tiab] OR groups [tiab]) NOT (animals [mh] NOT humans [mh])
