# Supplementary figures and images for: Impact of anesthetic agents on the amount of bleeding during dilatation and evacuation: A systematic review and meta-analysis
Source: PLoS One. 2021 Dec 22;16(12):e0261494. doi: 10.1371/journal.pone.0261494 (PMC8694452; doi:10.1371/journal.pone.0261494)

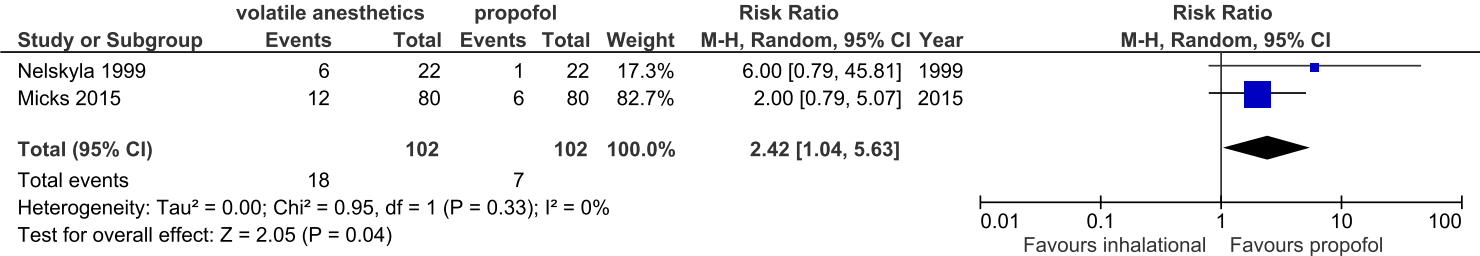

Supplement: S1 Fig — (PDF) [file pone.0261494.s003.pdf]

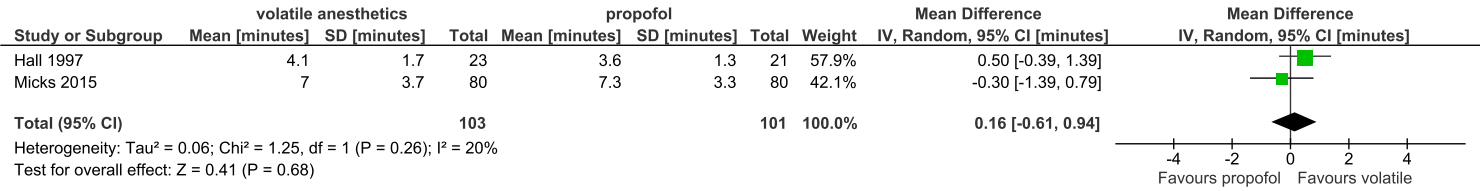

Supplement: S2 Fig — (PDF) [file pone.0261494.s004.pdf]

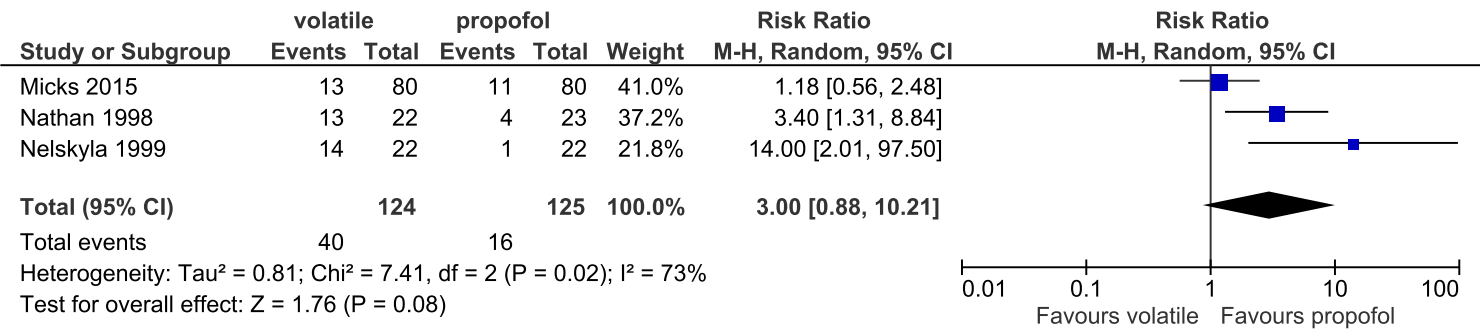

Supplement: S3 Fig — (PDF) [file pone.0261494.s005.pdf]
