# Supplementary material for: Impact of anesthetic agents on the amount of bleeding during dilatation and evacuation: A systematic review and meta-analysis
Source: PLoS One. 2021 Dec 22;16(12):e0261494. doi: 10.1371/journal.pone.0261494 (PMC8694452; doi:10.1371/journal.pone.0261494)
Supplement: S1 Table — (PDF) [file pone.0261494.s006.pdf]

Summary of findings:

Volatile anaesthetics compared to propofol in patients undergoing dilatation and evacuation

Patient or population: patients undergoing dilatation and evacuation
Setting:
Intervention: volatile anaesthetics
Comparison: propofol

Outcomes	**Anticipated absolute effects*** (95% CI)		Relative effect (95% CI)	№ of participants (studies)	Certainty of the evidence (GRADE)	Comments
	**Risk with propofol**	**Risk with volatile anaesthetics**				
amount of bleeding	The mean amount of bleeding was **163.9** mL	MD **164.7 mL higher** (43.6 higher to 285.7 higher)	-	343 (4 RCTs)	⨁◯◯◯ VERY LOW [a,b,c,d]	

***The risk in the intervention group** (and its 95% confidence interval) is based on the assumed risk in the comparison group and the **relative effect** of the intervention (and its 95% CI).

CI: Confidence interval; **MD:** Mean difference

GRADE Working Group grades of evidence
High certainty: We are very confident that the true effect lies close to that of the estimate of the effect
Moderate certainty: We are moderately confident in the effect estimate: The true effect is likely to be close to the estimate of the effect, but there is a possibility that it is substantially different
Low certainty: Our confidence in the effect estimate is limited: The true effect may be substantially different from the estimate of the effect
Very low certainty: We have very little confidence in the effect estimate: The true effect is likely to be substantially different from the estimate of effect

Explanations

a. Only one trial was considered to be at low risk of bias
b. I-squared was high.
c. 95% CI was wide.
d. Possibility of publication bias could not be denied.
