# Supplementary material for: Impact of anesthetic agents on the amount of bleeding during dilatation and evacuation: A systematic review and meta-analysis
Source: PLoS One. 2021 Dec 22;16(12):e0261494. doi: 10.1371/journal.pone.0261494 (PMC8694452; doi:10.1371/journal.pone.0261494)
Supplement: S1 Protocol — (PDF) [file pone.0261494.s007.pdf]

National Institute for Health Research

University of York
Centre for Reviews and Dissemination

Systematic review

Please complete all mandatory fields below (marked with an asterisk *) and as many of the non-mandatory fields as you can then click *Submit* to submit your registration. You don't need to complete everything in one go, this record will appear in your *My PROSPERO* section of the web site and you can continue to edit it until you are ready to submit. Click *Show help* below or click on the icon
to see guidance on completing each section.
This record cannot be edited because it has been rejected

1. * Review title.

Give the working title of the review, for example the one used for obtaining funding. Ideally the title should state succinctly the interventions or exposures being reviewed and the associated health or social problems. Where appropriate, the title should use the PI(E)COS structure to contain information on the Participants, Intervention (or Exposure) and Comparison groups, the Outcomes to be measured and Study designs to be included.

Does anesthetic agent affect the difficulty of dilatation and curretage; a systematic review and meta-analysis

2. Original language title.

For reviews in languages other than English, this field should be used to enter the title in the language of the review. This will be displayed together with the English language title.

3. * Anticipated or actual start date.

Give the date when the systematic review commenced, or is expected to commence.
01/05/2019

4. * Anticipated completion date.

Give the date by which the review is expected to be completed.
30/06/2019

5. * Stage of review at time of this submission.

Indicate the stage of progress of the review by ticking the relevant Started and Completed boxes. Additional information may be added in the free text box provided.
Please note: Reviews that have progressed beyond the point of completing data extraction at the time of initial registration are not eligible for inclusion in PROSPERO. Should evidence of incorrect status and/or completion date being supplied at the time of submission come to light, the content of the PROSPERO record will be removed leaving only the title and named contact details and a statement that inaccuracies in the stage of the review date had been identified.
This field should be updated when any amendments are made to a published record and on completion and publication of the review. If this field was pre-populated from the initial screening questions then you are not able to edit it until the record is published.

The review has not yet started: No

Review stage	Started	Completed
Preliminary searches	Yes	No
Piloting of the study selection process	No	No
Formal screening of search results against eligibility criteria	No	No
Data extraction	No	No
Risk of bias (quality) assessment	No	No
Data analysis	No	No

Provide any other relevant information about the stage of the review here (e.g. Funded proposal, protocol not yet finalised).

6. * Named contact.

The named contact acts as the guarantor for the accuracy of the information presented in the register record.

Hiromasa Kawakami

Email salutation (e.g. "Dr Smith" or "Joanne") for correspondence:

Dr Kawakami

7. * Named contact email.

Give the electronic mail address of the named contact.

hiromasa.kawakami@nifty.com

8. Named contact address

Give the full postal address for the named contact.

Department of Anesthesiology, Yokohama City University Medical Center, 4-57 Urafunecho Minamiku,

Yokohama Japan

9. Named contact phone number.

Give the telephone number for the named contact, including international dialling code.

+81-452615656

10. * Organisational affiliation of the review.

Full title of the organisational affiliations for this review and website address if available. This field may be completed as 'None' if the review is not affiliated to any organisation.

Yokohama City University Medical Center

Organisation web address:

11. * Review team members and their organisational affiliations.

Give the title, first name, last name and the organisational affiliations of each member of the review team. Affiliation refers to groups or organisations to which review team members belong.

Dr Hiromasa Kawakami. Yokohama City University Medical Center
Dr Hitoshi Sato. Yokohama City University Medical Center
Dr Takahiro Mihara. Yokohama City University

12. * Funding sources/sponsors.

Give details of the individuals, organizations, groups or other legal entities who take responsibility for initiating, managing, sponsoring and/or financing the review. Include any unique identification numbers assigned to the review by the individuals or bodies listed.

departmental funding

13. * Conflicts of interest.

List any conditions that could lead to actual or perceived undue influence on judgements concerning the main topic investigated in the review.

None

14. Collaborators.

Give the name and affiliation of any individuals or organisations who are working on the review but who are not listed as review team members.

15. * Review question.

State the question(s) to be addressed by the review, clearly and precisely. Review questions may be specific or broad. It may be appropriate to break very broad questions down into a series of related more specific questions. Questions may be framed or refined using PI(E)COS where relevant.

Does propofol reduce bleeding during dilatation and curretage and make the procedure easier?

16. * Searches.

Give details of the sources to be searched, search dates (from and to), and any restrictions (e.g. language or publication period). The full search strategy is not required, but may be supplied as a link or attachment.

We will search the following electronic bibliographic database: MEDLINE, EMBASE, Cochrane Central

Register of Controlled Trials (CENTRAL), Web of Science. We will also search trial registration site such as

ClinicalTrials.gov and University Medical Information Network Trial Registry.

The search strategy will include only terms relating the procedure and the intervention. The search strategy

for MEDLINE is uploaded. The search term will be adapted for use with other bibliographic databases.

There will be no language restriction. Studies published before the date the search are run will be sought.

17. URL to search strategy.

Give a link to a published pdf/word document detailing either the search strategy or an example of a search strategy for a specific database if available (including the keywords that will be used in the search strategies), or upload your search strategy.Do NOT provide links to your search results.

https://www.crd.york.ac.uk/PROSPEROFILES/120873_STRATEGY_20190429.pdf

Alternatively, upload your search strategy to CRD in pdf format. Please note that by doing so you are consenting to the file being made publicly accessible.

Yes I give permission for this file to be made publicly available

18. * Condition or domain being studied.

Give a short description of the disease, condition or healthcare domain being studied. This could include health and wellbeing outcomes.

patients who are undergoing dilatation and curretage

19. * Participants/population.

Give summary criteria for the participants or populations being studied by the review. The preferred format includes details of both inclusion and exclusion criteria.

patients who are undergoing dilatation and curretage

20. * Intervention(s), exposure(s).

Give full and clear descriptions or definitions of the nature of the interventions or the exposures to be reviewed.

Administering propofol for maintenance of general anesthesia

21. * Comparator(s)/control.

Where relevant, give details of the alternatives against which the main subject/topic of the review will be compared (e.g. another intervention or a non-exposed control group). The preferred format includes details of both inclusion and exclusion criteria.

Administering volatile agent for maintenance of anesthesia.

22. * Types of study to be included.

Give details of the types of study (study designs) eligible for inclusion in the review. If there are no restrictions on the types of study design eligible for inclusion, or certain study types are excluded, this should be stated. The preferred format includes details of both inclusion and exclusion criteria.

Randomised control studies will be included.

23. Context.

Give summary details of the setting and other relevant characteristics which help define the inclusion or exclusion criteria.

24. * Main outcome(s).

Give the pre-specified main (most important) outcomes of the review, including details of how the outcome is defined and measured and when these measurement are made, if these are part of the review inclusion criteria.

Blood loss

Timing and effect measures

During the procedure

25. * Additional outcome(s).

List the pre-specified additional outcomes of the review, with a similar level of detail to that required for main outcomes. Where there are no additional outcomes please state ‘None’ or ‘Not applicable’ as appropriate to the review

Surgical duration, subjective measurement of surgical difficulty, adverse events such as postoperative

nausea and vomiting, length of post anesthesia care unit stay or duration until hospital discharge.

Timing and effect measures

During and after the surgery

26. * Data extraction (selection and coding).

Give the procedure for selecting studies for the review and extracting data, including the number of researchers involved and how discrepancies will be resolved. List the data to be extracted.

Titles and abstracts of the studies retrieved using the search strategy and those from additional sourcesa will be screened independently by two review authors to identify studies that potentially meet inclusion criteria outlined above. The full text of these potentially aligible studies will be retrieved and independently assessed for eligibility by two review authors. Any disagreement between them over eligiblity of particular studies will be resolved through discussion.

A standardised, pre-piloted form will be used to extract data from the included studies for assessment of study quality and evidence synthesis. Extracted information will include: participant demographics and baseline characteristics; details of intervention and control condition (used inhalational anesthetic agent); recruitment and study completion rates; amount of bleeding during procedure, surgical duration, subjective measurement of surgical difficulty, the number of patients with adverse events; information for assessment of the risk of bias.

The two review authors will extract data independently, discrepancies will be identified and resolved through discussion. Missing data will be requested from study authors.

27. * Risk of bias (quality) assessment.

State whether and how risk of bias will be assessed (including the number of researchers involved and how discrepancies will be resolved), how the quality of individual studies will be assessed, and whether and how this will influence the planned synthesis.

Two review authos will independently assess the risk of bias in included studies by considering the following characteristics using Cochrane risk of bias tool:

Random sequence generation

Allocation concealment

Blinding of participant and personnel

Blinding of outcome assessor

incomplete outcome data

Selective outcome reporting

Other bias

Disagreement between the review authors over the risk of bias in particular studies will be resolved through

discussion

28. * Strategy for data synthesis.

Give the planned general approach to synthesis, e.g. whether aggregate or individual participant data will be used and whether a quantitative or narrative (descriptive) synthesis is planned. It is acceptable to state that a quantitative synthesis will be used if the included studies are sufficiently homogenous.

We compare the amount of bleeding and surgical duration with mean difference. We compare the surgical difficulies with risk ratio. We will summarize the mean difference or risk ratio with 95% confidence interval. If the 95% CI for mean difference included 0 for mean difference, or the 95% CI for risk ratio include 1 for risk ratio, we consider the difference not to be statistically significant. We use random effect model to combine the result. heterogeneity is quantified with I^2 statistic. Forest plot is used to graphically represent and evaluate the effect of treatment.

Small study effect is assessed useing funnel plot and Egger's regression asymmetry test when 10 or more studies are included. The test is considered positive if p is less than 0.1 in regression asymmetry test.

Sensitivity analysis are performed for primary outcomes according to the risk of bias (low vs high or unclear).

For primary outcomes, Trial Sequential Analysis (TSA) is performed to correcto for random error and repetitive testing of accumulating and sparse data. TSA monitoring boundaries (i.e. monitoring boundaries of meta-analysis) and required information size are quantified. Adjusted CIs are calculated. Risk of type 1 error is maintained at 5% with a power of 90%. The reduction of bleeding by 50 mL or surgical duration of 5 min was considered clinically meaningful.

29. * Analysis of subgroups or subsets.

Give details of any plans for the separate presentation, exploration or analysis of different types of participants (e.g. by age, disease status, ethnicity, socioeconomic status, presence or absence or co-morbidities); different types of intervention (e.g. drug dose, presence or absence of particular components of intervention); different settings (e.g. country, acute or primary care sector, professional or family care); or different types of study (e.g. randomised or non-randomised).

30. * Type and method of review.

Select the type of review and the review method from the lists below. Select the health area(s) of interest for your review.

Type of review

Cost effectiveness
No

Diagnostic
No

Epidemiologic
No

Individual patient data (IPD) meta-analysis
No

Intervention
No

Meta-analysis
Yes

Methodology
No

Narrative synthesis
No

Network meta-analysis
No

Pre-clinical
No

Prevention
No

Prognostic
No

Prospective meta-analysis (PMA)
No

Review of reviews
No

Service delivery
No

Synthesis of qualitative studies
No

Systematic review
Yes

Other
No

Health area of the review

Alcohol/substance misuse/abuse
No

Blood and immune system
No

Cancer
No

Cardiovascular
No

Care of the elderly
No

Child health
No

Complementary therapies
No

Crime and justice
No

Dental
No

Digestive system

No

Ear, nose and throat
No

Education
No

Endocrine and metabolic disorders
No

Eye disorders
No

General interest
No

Genetics
No

Health inequalities/health equity
No

Infections and infestations
No

International development
No

Mental health and behavioural conditions
No

Musculoskeletal
No

Neurological
No

Nursing
No

Obstetrics and gynaecology
No

Oral health
No

Palliative care
No

Perioperative care
No

Physiotherapy
No

Pregnancy and childbirth
No

Public health (including social determinants of health)
No

Rehabilitation
No

Respiratory disorders
No

Service delivery
No

Skin disorders
No

Social care
No

Surgery
Yes

Tropical Medicine
No

Urological
No

Wounds, injuries and accidents
No

Violence and abuse
No

31. Language.

Select each language individually to add it to the list below, use the bin icon to remove any added in error.
English

There is not an English language summary

32. Country.

Select the country in which the review is being carried out from the drop down list. For multi-national collaborations select all the countries involved.
Japan

33. Other registration details.

Give the name of any organisation where the systematic review title or protocol is registered (such as with The Campbell Collaboration, or The Joanna Briggs Institute) together with any unique identification number assigned. (N.B. Registration details for Cochrane protocols will be automatically entered). If extracted data will be stored and made available through a repository such as the Systematic Review Data Repository (SRDR), details and a link should be included here. If none, leave blank.

34. Reference and/or URL for published protocol.

Give the citation and link for the published protocol, if there is one

Give the link to the published protocol.

Alternatively, upload your published protocol to CRD in pdf format. Please note that by doing so you are consenting to the file being made publicly accessible.

No I do not make this file publicly available until the review is complete

Please note that the information required in the PROSPERO registration form must be completed in full even if access to a protocol is given.

35. Dissemination plans.

Give brief details of plans for communicating essential messages from the review to the appropriate audiences.

we plan to publish in anesthesia journal.

Do you intend to publish the review on completion?

Yes

36. Keywords.

Give words or phrases that best describe the review. Separate keywords with a semicolon or new line. Keywords will help users find the review in the Register (the words do not appear in the public record but are included in searches). Be as specific and precise as possible. Avoid acronyms and abbreviations unless these are in wide use.

dilatation and curretage

anesthetic agent

propofol

sevoflurane

desflurane

isoflurane

general anesthesia

37. Details of any existing review of the same topic by the same authors.

Give details of earlier versions of the systematic review if an update of an existing review is being registered, including full bibliographic reference if possible.

none

38. * Current review status.

Review status should be updated when the review is completed and when it is published. For newregistrations the review must be Ongoing.
Please provide anticipated publication date

Review_Ongoing

39. Any additional information.

Provide any other information the review team feel is relevant to the registration of the review.

40. Details of final report/publication(s).

This field should be left empty until details of the completed review are available.

Give the link to the published review.
